# The Meetings at Old Point Comfort, 1897

**Published:** 1897-09

**Authors:** 


### EDITORIAL.

### The Meetings at Old Point Comfort, 1897.

The annual meeting of the National Association of Dental Faculties was held at Old Point Comfort on July 30th, 31st and August 2d.

There was almost a full delegation of colleges having membership in this body. The meeting was a harmonious one. There were many points presented pertaining to college management and upon these, as usual, there was some diversity of opinion. Free and frank discussion was had, and in every instance such conclusions and decisions were reached as seemed to be in the main satisfactory to all. Some new features of work were provided for future meetings, among which may be mentioned the presentation of papers relating to college work and management with a view of bringing out the best methods of work and college control.

A most excellent paper was read on teaching dental anatomy by Dr. W. C. Barrett. This paper was highly interesting and instructive to many, if not all present.

(A synopsis of the proceedings will be found in the present number of the REGISTER.)

Dr. Truman W. Brophy was made President for the coming year. The official proceedings will be published in pamphlet form within the next few weeks, a copy of which can be obtained from the Secretary, Dr. J. H. Kennerly, St. Louis, Mo.

THE TECHNIQUE ASSOCIATION.

This body did not hold its meeting at this time, the regular meeting having been postponed to a future time, and place yet to be designated.

### THE NATIONAL ASSOCIATION OF DENTAL EXAMINERS.

This body held its annual meeting at the same time as the Faculties Association. The work done by this body was of much interest to the profession and dental colleges as well. The effort of this association throughout its meetings was with a view to obtain a more harmonious relationship with the Dental Faculties Association, and this effort was in a good degree successful. The proceedings of this body will be published, in the near future, in this journal.

### THE AMERICAN DENTAL ASSOCIATION AND THE SOUTHERN DENTAL ASSOCIATION.

The meetings and work of these two bodies constituted an important feature of the entire occasion. Efforts have been in operation for the last two or three years, looking to a union of these bodies. A program, constitution and by-laws, preparatory to this result, had been formulated within the last year, and so well had this work been done that no difficulty was experienced in the consummation of the desired object, upon the plan proposed; there was entire harmony in both associations, and the union of the two bodies was effected without a jar, and a new organization, under the name of the National Dental Association, was effected.

The American Association, having completed its work and closed up all its business affairs, adjourned *sine die*—passed out of existence.

The Southern Dental Association, having within the last year or two planned some special work, which it deemed desirable to perform, still continues its organization, and which work when completed, this body will, as the American did, close its career.

We now have but one National Association, and this will undoubtedly be made so in the full meaning of the term, by the combined efforts of all the representative members of the profession in the United States. Every State society should become an active auxilliary of this central body. Indeed, this is in a good degree provided for in the new constitution.

This union is an epoch in the dental profession of this country, from which doubtless a new impulse will be started that will ultimate in greater growth than has at any time in the past been realized. It is to be hoped that every member will work for the attainment of this end. If the best results are to be attained, they can only be reached by the earnest, determined effort of every individual. Dr. T. Fillebrown, of Boston, Mass., was elected president of the new organization.

